# Timing it right: dephosphorylation of a key transcription factor constrains the tapetal transcriptional cascade for timely pollen development

**DOI:** 10.1093/plcell/koag294

**Published:** 2026-09-23

**Authors:** Pablo González-Suárez, Cecilia Lara-Mondragón

**Affiliations:** Assistant Features Editor, The Plant Cell, American Society of Plant Biologists; Department of Developmental Genetics, Centre for Plant Molecular Biology (ZMBP), Eberhard Karls University, Tuebingen D-72076, Germany; Department of Developmental Genetics, Centre for Plant Molecular Biology (ZMBP), Eberhard Karls University, Tuebingen D-72076, Germany

Once made, some decisions are impossible to undo. For plants, the significance of certain developmental choices weighs heavily: processes like germination, flowering, and seed set must be finely timed to maximize survival. During tissue development, the need for temporal precision is apparent as new cell types must emerge at the right time and place. In pollen development, microspores are initially surrounded by the tapetum, a supporting somatic layer that secretes nutrients and components necessary for pollen grain formation. Later, the tapetum undergoes programmed cell death, releasing its final cargo of support substances, including precursors of the pollen cell wall. Timely development of the tapetum is crucial for pollen fertility, with precocious or delayed tapetal activity leading to severe sterility (Lou et al. 2018). As such, activation of the tapetal program is tightly controlled by a complex transcriptional network of feed-forward and positive feedback loops. While these reinforce the initial commitment to tapetal development, it is less understood how excessive transcriptional activation is prevented to ensure pollen wall homeostasis.

Curious about the self-reinforcing nature of the tapetal network, Zesen Lai and colleagues (Lai et al. 2026) set out to dissect the mechanisms that enable its timely inactivation. Specifically, they focused on the transcription factor bHLH089, a known key actor in the network which amplifies the transcriptional output during early tapetum development (Zhu et al. 2015; Cui et al. 2016). Their genetic analysis showed that both reduced and excessive bHLH089 accumulation disrupt pollen wall integrity leading to fertility defects, suggesting that upstream mechanisms are normally enforced to restrain bHLH089 activity.

In the search for regulators of bHLH089, the authors used a combination of genetic screens and biochemical assays that identified 2 interaction partners, the protein phosphatases PP2A-C1 and PP2A-C5. Intriguingly, they noted that co-expression of bHLH089 with these phosphatases led to changes in its subcellular localization. While bHLH089 alone localizes to the nucleus, co-expression with SUPPRESSOR OF BRI1 (SBI1), a regulator of PP2A assembly and activity, significantly reduced bHLH089 nuclear retention. Together, this suggests that SBI1 and PP2A-C1/PP2A-C5 form a complex that controls bHLH089 subcellular distribution.

To explore the kinetics of the interactions between SBI1, PP2As, and bHLH089, the authors analyzed their gene expression patterns at different stages of anther development. Consistent with a potential role in restricting bHLH089, the *SBI1-PP2A-C*s module showed strongest expression toward later developmental stages, shortly after *bHLH089* has surpassed its expression peak (Fig. 1). Examination of transgenic lines that overexpress both *bHLH089* and either of the phosphatases confirmed this negative regulation, highlighting that PP2A-Cs restrict bHLH089 output at the protein accumulation level. Accordingly, transcriptional activation of the tapetal program downstream of bHLH089 is impaired in mutants and transgenics where an excess of the SBI1-PP2A-C module compromises bHLH089 nuclear retention.

**Figure 1:**
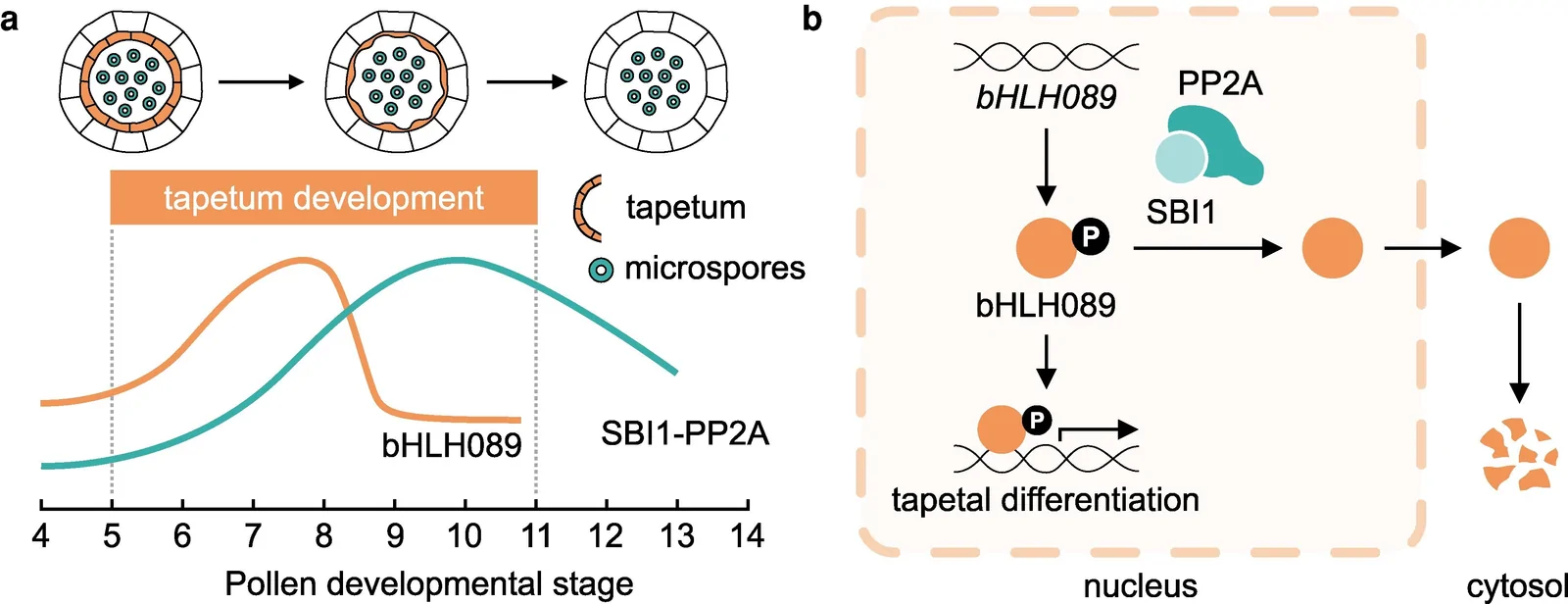
PP2A-driven dephosphorylation limits nuclear retention of bHLH089 for proper pollen development. Lai and colleagues demonstrate that a SB1-PP2a phosphatase module peaks during late tapetal development to attenuate bHLH089 activity (a). The work suggests a model whereby PP2A-driven dephosphorylation of bHLH089 leads to nuclear export, allowing for timely inactivation of tapetal transcriptional cascades (b). Figure credit: P. González-Suárez.

Lastly, the authors asked whether the phosphorylation status of specific bHLH089 residues modulates its nuclear retention. Through computational prediction, they identified 2 serine residues potentially susceptible to PP2A phosphorylation and used genetic analyses to validate their impact on bHLH089 localization. Replacing either of the 2 serines with phospho-dead variants disrupted nuclear accumulation of the transcription factor, while phospho-mimic versions of the residues maintained strict nuclear localization. These data further highlight the potential of dephosphorylation of bHLH089 as a molecular mechanism to drive nuclear export and hence attenuate the downstream tapetal cascade.

Together, the findings of Lai and coauthors (Lai et al. 2026) demonstrate that the activity of bHLH089 is constrained to a specific time window through a phosphatase-driven module. Their observations place PP2A phosphatases in the spotlight as key regulators of protein subcellular accumulation and transcription factor competency. Beyond pollen development, their work also highlights a novel buffering mechanism that regulates the kinetics of bHLH transcription factor activity. Notably, the identified phosphatase-sensitive residues of bHLH089 are highly conserved across the Brassicaceae family. Interestingly, this might suggest that this mechanism plays broader roles in development through timing the protein activity and safeguarding key developmental phase transitions.

## Recent related articles in *The Plant Cell*

- Hua et al. (2025) characterized a central role for the bHLH transcription factor DTT1 as part of a heterodimer complex that drives tapetum cell fate in barley.
- Wu et al. (2026) found that post-translational O-glycosylation stabilizes the NAC transcription factor SlNOR to control tomato fruit ripening.
- Zhao et al. (2026) uncovered that tissue and temporal specificity in alternative polyadenylation functions as a cotranscriptional mechanism during pollen development.

## Data Availability

No new data were generated or analyzed in support of this research.
